# CD3 directed bispecific antibodies induce increased lymphocyte–endothelial cell interactions in vitro

**DOI:** 10.1054/bjoc.1999.0945

**Published:** 2000-01-18

**Authors:** G Molema, J W Cohen Tervaert, B J Kroesen, W Helfrich, D K F Meijer, L F M H de Leij

**Affiliations:** Department of Pathology and Lab Medicine, Groningen University Institute for Drug Exploration (GUIDE), Laboratory for Tumor Immunology, Hanzeplein 1, Groningen, GZ, 9713, The Netherlands; Department of Pharmacokinetics and Drug Delivery, University Center for Pharmacy, Ant. Deusinglaan 1, Groningen, AV, 9713, The Netherlands; Department of Clinical Immunology, Hanzeplein 1, Groningen, GZ, 9713, The Netherlands

**Keywords:** immunotherapy, CD3 directed bispecific antibody, PBMC, adhesion, transendothelial migration, in vitro

## Abstract

Bispecific antibody (BsMAb) BIS-1 has been developed to redirect the cytolytic activity of cytotoxic T lymphocytes (CTL) to epithelial glycoprotein-2 (EGP-2) expressing tumour cells. Intravenous administration of BIS-1 F(ab′)_2_to carcinoma patients in a phase I/II clinical trial, caused immunomodulation as demonstrated by a rapid lymphopenia prior to a rise in plasma tumour necrosis factor-α and interferon-γ levels. Yet, no lymphocyte accumulation in the tumour tissue and no anti-tumour effect could be observed. These data suggest a BsMAb-induced lymphocyte adhesion to blood vessel walls and/or generalized redistribution of the lymphocytes into tissues. In this study, we describe the effects of BIS-1 F(ab′)_2_binding to peripheral blood mononuclear cells (PBMC) on their capacity to interact with resting endothelial cells in vitro. Resting and pre-activated PBMC exhibited a significant increase in adhesive interaction with endothelial cells when preincubated with BIS-1 F(ab′)_2_, followed by an increase in transendothelial migration (tem). Binding of BIS-1 F(ab′)_2_to PBMC affected the expression of a number of adhesion molecules involved in lymphocyte adhesion/migration. Furthermore, PBMC preincubated with BIS-1 F(ab′)_2_induced the expression of endothelial cell adhesion molecules E-selectin, VCAM-1 and ICAM-1 during adhesion/tem. These phenomena were related to the CD3 recognizing antibody fragment of the BsMAb and dependent on lymphocyte–endothelial cell contact. Possibly, in patients, the BIS-1 F(ab′)_2_infusion induced lymphopenia is a result of generalized activation of endothelial cells, leading to the formation of a temporary sink for lymphocytes. This process may distract the lymphocytes from homing to the tumour cells, and hence prevent the occurrence of BIS-1 F(ab′)_2_– CTL-mediated tumour cell lysis. © 2000 Cancer Research Campaign


*British Journal of Cancer* (2000) **82**(2), 472–479
© 2000 Cancer Research Campaign
Article no. bjoc.1999.0945

### CD3 directed bispecific antibodies induce increased lymphocyte–endothelial cell interactions in vitro

**G Molema[1,2], JW Cohen Tervaert[3], BJ Kroesen[1], W Helfrich[1], DKF Meijer[2] and LFMH de Leij[1]**

Groningen University Institute for Drug Exploration (GUIDE), [1]Department of Pathology and Lab Medicine, Laboratory for Tumor Immunology, Hanzeplein 1, 9713 GZ Groningen, The Netherlands; [2]Department of Pharmacokinetics and Drug Delivery, University Center for Pharmacy, Ant. Deusinglaan 1, 9713 AV Groningen, The Netherlands; [3]Department of Clinical Immunology, Hanzeplein 1, 9713 GZ Groningen, The Netherlands

**Summary** Bispecific antibody (BsMAb) BIS-1 has been developed to redirect the cytolytic activity of cytotoxic T lymphocytes (CTL) to epithelial glycoprotein-2 (EGP-2) expressing tumour cells. Intravenous administration of BIS-1 F(ab′)$_2$ to carcinoma patients in a phase I/II clinical trial, caused immunomodulation as demonstrated by a rapid lymphopenia prior to a rise in plasma tumour necrosis factor-$\alpha$ and interferon-$\gamma$ levels. Yet, no lymphocyte accumulation in the tumour tissue and no anti-tumour effect could be observed. These data suggest a BsMAb-induced lymphocyte adhesion to blood vessel walls and/or generalized redistribution of the lymphocytes into tissues. In this study, we describe the effects of BIS-1 F(ab′)$_2$ binding to peripheral blood mononuclear cells (PBMC) on their capacity to interact with resting endothelial cells in vitro. Resting and pre-activated PBMC exhibited a significant increase in adhesive interaction with endothelial cells when preincubated with BIS-1 F(ab′)$_2$, followed by an increase in transendothelial migration (tem). Binding of BIS-1 F(ab′)$_2$ to PBMC affected the expression of a number of adhesion molecules involved in lymphocyte adhesion/migration. Furthermore, PBMC preincubated with BIS-1 F(ab′)$_2$ induced the expression of endothelial cell adhesion molecules E-selectin, VCAM-1 and ICAM-1 during adhesion/tem. These phenomena were related to the CD3 recognizing antibody fragment of the BsMAb and dependent on lymphocyte–endothelial cell contact. Possibly, in patients, the BIS-1 F(ab′)$_2$ infusion induced lymphopenia is a result of generalized activation of endothelial cells, leading to the formation of a temporary sink for lymphocytes. This process may distract the lymphocytes from homing to the tumour cells, and hence prevent the occurrence of BIS-1 F(ab′)$_2$ – CTL-mediated tumour cell lysis. © 2000 Cancer Research Campaign

**Keywords:** immunotherapy; CD3 directed bispecific antibody; PBMC; adhesion; transendothelial migration; in vitro

In recent years a number of immunotherapeutical strategies have been developed for the treatment of solid tumours and metastatic disease. Bispecific antibodies (BsMAb) that cross-link the CD3/T-cell receptor (TcR) complex on T lymphocytes and tumour-associated antigens have been shown to effectively redirect the cytolytic activity of CTLs to tumour cells (de Gast et al, 1995; Demanet et al, 1996; Renner et al, 1996). In our laboratory we developed BIS-1 BsMAb which is able to cross-link the CD3/TcR complex on CTLs and the tumour-associated antigen EGP-2 on carcinoma cells (de Leij et al, 1994). Intraperitoneal injection of in vitro activated, BIS-1 IgG-coated autologous T-cells into patients with malignant ascites or pleural exudates caused a significant anti-tumour effect and a strong local inflammatory reaction (Kroesen et al, 1993). In a phase I/II trial, intravenous administration of BIS-1 F(ab′)$_2$ to renal cell carcinoma patients with disseminated disease during standard subcutaneous (s.c.). IL-2 treatment also induced immune cell activation (Kroesen et al, 1994). There was, however, no evidence that BIS-1-coated lymphocytes distributed into tumour tissue, and so far no notable anti-tumour responses have been observed (Nieken et al, unpublished observations).

One important observation in clinical studies of systemic administration of CD3 directed BsMAbs in general, and BIS-1 F(ab′)$_2$ in specific, for the treatment of metastatic disease is the strong lymphopenia starting as early as 30 min after initiation of BsMAb infusion (de Gast et al, 1995; Janssen et al, 1995). Success of BsMAb therapy is dependent on the contact between immune effector cells and tumour cells. It is therefore important to understand the cause(s) of lymphocyte disappearance from the peripheral blood upon BIS-1 F(ab′)$_2$ infusion and the lack of distribution into the tumour tissue. Understanding the underlying mechanism(s) may lead to new strategies that can selectively target CTLs to the tumour tissue and improve therapeutic outcome (Molema et al, 1997, 1998*b*). Possibly, the lymphopenia observed during intravenous (i.v.) BIS-1 F(ab′)$_2$ administration is the result of a (temporary) increased adhesiveness of lymphocytes to the blood vessel walls. Subsequently, lymphocytes can extravasate, migrate into organ parenchyma and re-appear in the circulation via the lymphatic system. The present study describes the effects of binding of CD3- versus CD5-directed BsMAbs to peripheral blood mononuclear cells (PBMC) on interactions between PBMC and resting endothelial cell monolayers, the latter representing the status of the majority of endothelial cells in the body. Important for the interactions between leucocytes and endothelial cells (Figure 1) are the cell adhesion molecules sialyl Lewis$^x$/E- and L-selectin, VLA-4/VCAM-1 and LFA-1/ICAM-1 (Springer, 1994). We therefore also studied the expression of these cell adhesion molecules on PBMC after binding of BsMAb, and on endothelium during adhesion/transendothelial migration of BsMAb-coated PBMC.

*Received 12 April 1999*
*Revised 13 July 1999*
*Accepted 19 July 1999*

*Correspondence to:* LFMH De Leij

**Table 1** Summary of antibodies used for flow cytometric analysis of cell adhesion molecule expression

| Antibody name | Antigen recognized | Source |
|---|---|---|
| Anti-CD15s | CD15s, sialyl Lewis$^x$ | Pharmingen, San Diego, CA, USA |
| Leu8 | L-selectin | Becton Dickinson, San Jose, CA, USA |
| HP2/1 | VLA-4 | Dr M Sánchez-Madrid, Madrid, Spain |
| NKI.L16 | LFA-1, high affinity variant | Dr Y van Kooyk, Nijmegen, The Netherlands |
| NKI.L15 | LFA-1, total | Dr Y van Kooyk, Nijmegen, The Netherlands |
| anti-CD31 | CD31 (PECAM-1) | Dako A/S, Glostrup, Denmark |
| 24–31 (FITC) | CD40-L | Dr A Kashran, Leuven, Belgium |
| H18/7 | E-selectin | Dr MA Gimbrone Jr, Boston, MA, USA |
| E1/6 | VCAM-1 | Dr MA Gimbrone Jr |
| Hu5/3 | ICAM-1 | Dr MA Gimbrone Jr |
| Anti-CD3 (PE) | CD3 | Southern Biotechn. Assoc., Birmingham, AL, USA |
| Anti-CD4 (FITC) | CD4 | Immuno Quality Products, Groningen, The Netherlands |
| Anti-CD8 (PE) | CD8 | Immuno Quality Products |
| 1A29 | rat ICAM-1 | Dr Miyasaka, Osaka, Japan |

### MATERIALS AND METHODS

### Monoclonal antibodies

Primary monoclonal antibodies used in this study are summarized in Table 1. Fluorescein isothiocyanate (FITC)- labelled rabbit anti-mouse Ig F(ab′)$_2$ was obtained from Dako A/S (Glostrup, Denmark).

### Bispecific antibodies

Characteristics of the BsMAbs BIS-1 F(ab′)$_2$ (Kroesen et al, 1994), BIS-18 IgG (Kroesen et al, 1995) and SHR-1 IgG (Clark et al, 1989) used in this study are given in Table 2. SHR-1 was a kind gift of Dr M Clark, Cambridge, UK. Solutions of the BsMAbs used in the described assays were tested negative for lipopolysaccharide using the *Limulus* assay (Nodia/Chromogenics, Leiden, The Netherlands).

### Cells

*PBMC*

Human PBMC were obtained from heparinized blood by density centrifugation on Lymphoprep (Nycomed, Oslo, Norway). After washing, PBMC were resuspended in RPMI-1640 supplemented with 2% heat inactivated pooled human serum, 2 mM glutamine and 60 µg ml$^{-1}$ gentamycin (hereafter referred to as RPMI complete medium). PBMC kept overnight in RPMI complete medium were considered resting. PDB activation was achieved by culturing PBMC for 24 h at 5 ng ml$^{-1}$ phorbol 12,13-dibutyrate (PDB; Sigma Chemical Co., Zwijndrecht, The Netherlands) in

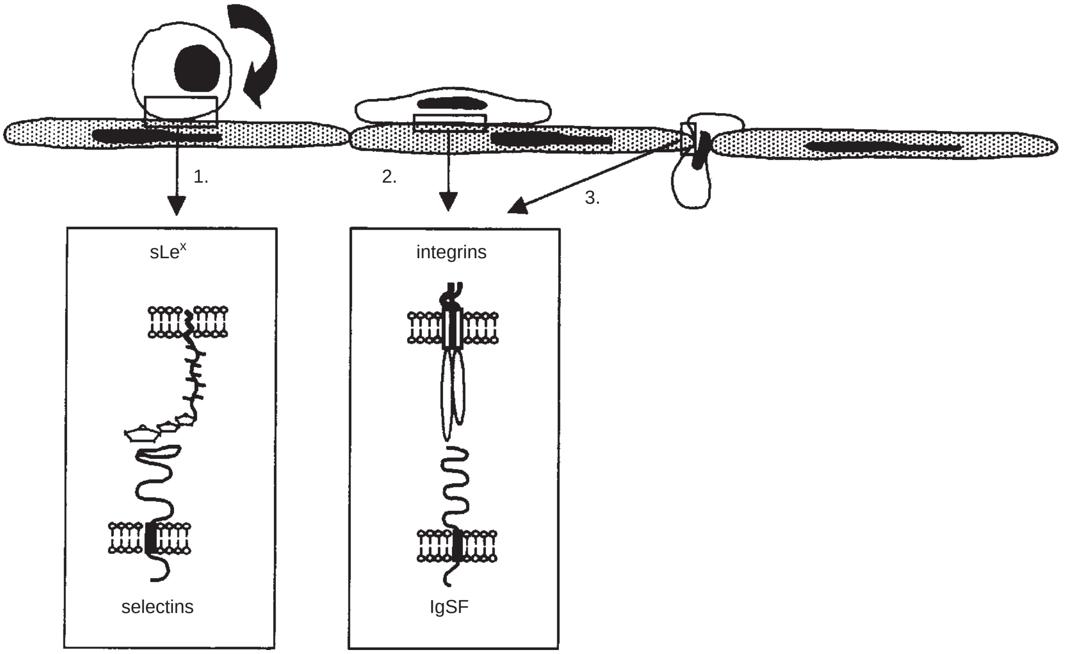

**Figure 1** Lymphocyte–endothelial cell interactions leading to lymphocyte extravasation into tissue. Tethering of the lymphocytes (1) is accomplished by interactions between selectin molecules on both lymphocytes and endothelial cells and their sialyl Lewis$^x$ (sLe$^x$) ligands. This is followed by firm adhesion and lymphocyte activation (2), and transendothelial migration (3). Adhesion, activation and migration are mediated by integrins VLA-4 and LFA-1 on lymphocytes, and their respective counterstructures VCAM-1 and ICAM-1 (both Immunoglobulin SuperFamily (IgSF) members), expressed on the endothelium. After transendothelial migration lymphocytes move into the tissue mediated by concerted actions of soluble factors such as chemoattractants, and integrins, among others

**Table 2** Characteristics of the bispecific antibodies (BsMAb) used in this study

| BsMAb | BsMAb format | Therapeutic use | T-cell recognition | | | Tumour cell recognition | | |
|---|---|---|---|---|---|---|---|---|
| | | | Antigen | Antibody name | Antibody isotype | Antigen | Antibody name | Antibody isotype |
| BIS-1 | $F(ab')_2$ | Renal cell carcinoma | CD3 | RIV-9 | Mouse IgG3 | EGP-2 | MOC31 | Mouse IgG1 |
| BIS-18 | IgG | (Not used) | CD5 | 83-P2E6 | Mouse IgG2b | EGP-2 | MOC31 | Mouse IgG1 |
| SHR-1 | IgG | Non-Hodgkin's lymphoma | CD3 | YTH12.5 | Rat IgG2b | CD19 | MG1CD19 | Mouse IgG1 |

RPMI complete medium (Oppenheimer-Marks et al, 1991). Interleukin (IL)-2 activated PBMC were cultured in the presence of 100 IU $ml^{-1}$ rec. huIL-2 (EuroCetus, Amsterdam, The Netherlands) for 5 days, during which the medium was refreshed after 3 days. Anti-CD3/IL-2-activated PBMC were obtained by culturing the cells in the presence of the mitogenic anti-CD3 MAb WT32 (Tax et al, 1983) for 3 days, washing and culturing for an additional 2 days in complete medium supplemented with 100 IU $ml^{-1}$ rec. huIL-2. All PBMC populations were cultured at 37°C in a humidified atmosphere containing 5% carbon dioxide.

Phenotyping of the PBMC populations showed that resting, PDB and IL-2-activated lymphocytes consisted of more than 75%, and anti-CD3/IL-2-activated lymphocytes of more than 98% CD3-positive T lymphocytes. These percentages as well as the CD4/CD8 ratio differed significantly between different donors (data not shown).

### *Endothelial cells*

Human umbilical vein endothelial cells (HUVEC) were isolated and cultured in the presence of 20% human serum and supplements as described by Mulder et al (1994). By routine flow cytometric phenotyping for CD31 the cells were found to be uniformly positive. HUVEC passages 2–4 were used for the studies presented.

### Determination of lymphocyte adhesion/transendothelial migration

Experiments to investigate the effects of BsMAb binding to PBMC on interactions with endothelium were performed as described (Molema et al, 1998*a*). Briefly, HUVEC were seeded on Vitrogen 100 collagen layers (Collagen Corporation, Palo Alto, CA, USA) at confluent cell density and cultured for 48 h. Precoating of PBMC with BsMAbs was performed for 45 min at RT at 1 µg $ml^{-1}$ BsMAb in RPMI complete medium, whereas uncoated PBMC were incubated in RPMI complete medium only. After extensive washing, both uncoated and BsMAb-coated PBMC were resuspended at $5–7 \times 10^5$ cells $ml^{-1}$ in RPMI complete medium. The adhesion/tem assay was started by applying 500 µl PBMC suspension per well. After incubation at 37°C, 5% carbon dioxide in a humidified atmosphere for 3 h, non-adherent PBMC were removed by extensive washing, adherent cells were detached by 0.05% trypsin/0.5 mM EDTA (ICN Biomedicals B.V., Zoetermeer, The Netherlands) treatment, and migrated cells by treatment with collagenase (2 mg $ml^{-1}$ in PBS, freshly prepared; collagenase was a kind gift of Knoll A/G, Ludwigshafen, Germany). Alternatively, HUVEC were cultured in the presence of 20% fetal calf serum (Biowhitaker, Brussels, Belgium). Under these conditions the adhesion/tem assays were performed using HUVEC cultured on gelatin-coated wells, an experimental setup frequently applied by others (Masinovsky et al, 1990; Thornhill et al, 1990). In this setup, adherent/migrated cells were released by incubation of the wells with trypsin/EDTA, and analysis of the total number of adherent plus migrated PBMC was performed. Results from both assays were comparable with respect to absolute numbers of adherent plus migrated PBMC and the effects of the BsMAbs. Adhesion/tem results shown are obtained with HUVEC cultured on collagen gels unless otherwise stated.

Flow cytometric determination of absolute cell numbers was performed using fluorosphere reagent (Immuno Check EPICS alignment fluorospheres or Flow Count fluorospheres from Coulter Corp., Hialeah, FL, USA) as described (Molema et al, 1998*a*).

### Flow cytometric analysis of adhesion molecule expression by PBMC and HUVEC

All incubations and wash steps were performed at 4°C. Cells were incubated with primary antibodies in phosphate-buffered saline (PBS) supplemented with 5% human pooled serum. When using anti-LFA-1 antibody NKI-L16 (Van Kooyk et al, 1991), all incubations were performed in $Ca^{2+}/Mg^{2+}$ free HBSS buffer (Gibco-BRL/Life Technologies). In the case of indirect fluorescence staining, detection was performed with FITC-labelled rabbit anti-mouse Ig $F(ab')_2$. Flow cytometric analysis was performed on a Coulter Elite Cytometer (Coulter Electronics, Hialeah, FL, USA). Ten thousand (PBMC) and 2000 (HUVEC) events were recorded and analysed with Winlist (V3.1) from Verity Software House, ME, USA.

### Statistical analysis

The effect of BsMAb binding to PBMC on PBMC adhesion and transendothelial migration was analysed statistically using the two-sided Student's *t*-test for unpaired samples with different standard deviation (s.d.) $P$-values of $< 0.05$ were considered to be significant at 95% confidence intervals.

### RESULTS

### Effect of PBMC activation status on adhesion/tem

The effects of PBMC activation on their subsequent interaction with resting HUVEC is shown in Figure 2. Resting PBMC exhibited low adhesive and migratory capacity. Activation of the PBMC with either IL-2 or PDB led to a three-to fourfold increase in both adhesion and tem. Anti-CD3/IL-2 activation of PBMC led to a

© 2000 Cancer Research Campaign

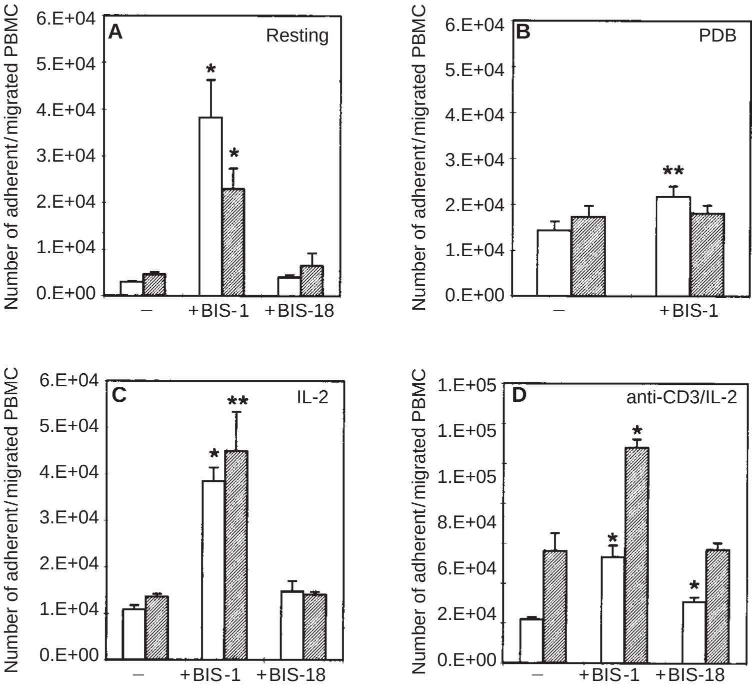

**Figure 2** Effect of PBMC activation status and BsMAb binding on PBMC adhesion/tem to/through monolayers of resting HUVEC. PBMC were activated as described in Materials and Methods. After pre-incubation of PBMC at RT for 45 min with 1 μg $ml^{-1}$ of either BIS-1F $(ab')_2$ or BIS-18, cells were subjected to the adhesion/tem assay. Open bars represent adherent, hatched bars migrated PBMC. Note the difference in scale in **D** as compared to **A, B** and **C**. Values are means ± s.d. of triplicate experiments. Statistically significant increases in adhesion or migration compared to PBMC not pre-incubated with BsMAb: * $P < 0.02$, ** $0.02 < P < 0.05$

sevenfold increase in lymphocyte adherence compared to resting PBMC. In contrast to the other activation routes, activation with anti-CD3/IL-2 equipped the PBMC with an enhanced migratory capacity as well.

### Effect of BsMAb binding to PBMC on PBMC adhesion/tem

### *Effect on PBMC adhesion*

In all PBMC populations studied, binding of BIS-1 $F(ab')_2$ to the cells significantly affected PBMC–endothelial cell interactions (Figure 2), albeit to a different extent. An irrelevant BsMAb, BIS-100 (anti-rat CD3/TcR × anti-EGP-2) did not affect adhesion and migration of PBMC (data not shown). The anti-CD5 directed BsMAb BIS-18 induced a slight (1.3- to 1.4-fold) increase in adhesion in all PBMC populations studied. These latter increases were, however, statistically not significant except for the effect on the adhesion of anti-CD3/IL-2 activated PBMC ($P = 0.01$).

### *Effect on PBMC migration*

For all PBMC populations except the PDB activated cells, tem of BIS-1 $F(ab')_2$ pre-incubated cells was significantly increased (Figure 2). The absolute numbers of migrated cells in these populations differed notably, possibly reflecting the differences in intrinsic migratory capacity of the various PBMC populations. Binding of BIS-18 did not exert an effect on the migration of the PBMC populations studied (Figure 2).

In different experiments, when using different PBMC and HUVEC isolates, the absolute numbers of adherent and migrated cells varied significantly between experiments. In all experiments, however, we observed similar increases in adhesion/tem of PBMC pre-incubated with BIS-1 $F(ab')_2$, but not BIS-18, as described above. The data presented are therefore representative of other experiments.

### Effect of BsMAb binding to PBMC on lymphocyte adhesion molecule expression

The increased in vitro adhesion/migration characteristics of resting PBMC preincubated with BIS-1 $F(ab')_2$ led us to determine the effect of BIS-1 $F(ab')_2$ and BIS-18 binding to PBMC on the expression of lymphocyte adhesion molecules now believed to play an important role in adhesion/tem. Most notably were the increased expression of CD15s and CD31, and decreased expression of L-selectin on PBMC preincubated with BIS-1 $F(ab')_2$ (Figure 3). BIS-18 binding to PBMC did not affect these particular adhesion molecules. Both BIS-1 $F(ab')_2$ and BIS-18 induced a rise in the expression of VLA-4 and high affinity LFA-1 (L16 epitope), whereas total LFA-1 (L15 epitope) expression slightly changed. This suggests that both BIS-1 $F(ab')_2$ and BIS-18 binding under the conditions used were able to activate PBMC, but to a different extent.

### Adhesion molecule expression of HUVEC after PBMC adhesion/tem

We next determined whether the adhesion molecule expression of HUVEC changed during the actual adhesion/tem process of resting PBMC preincubated with BIS-1 $F(ab')_2$. Figure 4 shows that the expression of especially E-selectin and VCAM-1

© 2000 Cancer Research Campaign 

markedly increased during the 3-h period in which the adhesion/tem of BIS-1 F(ab′)$_2$-coated PBMC took place, whereas ICAM-1 expression only moderately increased. No changes were observed after adhesion/tem of resting PBMC or PBMC preincubated with BIS-18 (data not shown). From this it was concluded that resting PBMC coated with BIS-1 F(ab′)$_2$ were able to activate HUVEC to express adhesion molecules during PBMC adhesion/tem.

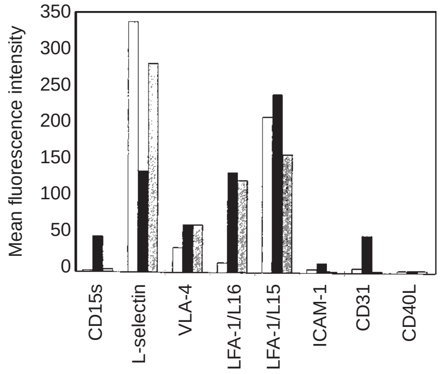

**Figure 3** Effect of BIS-1 F(ab′)$_2$ and BIS-18 binding to resting PBMC on the expression of lymphocyte cell surface markers as measured by flow cytometry. Open bars: PBMC; closed bars: PBMC preincubated with BIS-1 F(ab′)$_2$; hatched bars: PBMC preincubated with BIS-18. Both BsMAbs were present for 45 min at RT at 1.0 μg ml$^{-1}$ as described in Materials and Methods. For those antibodies used in an indirect immunofluorescence protocol, the mean fluorescence intensities were corrected for the signal obtained with the control antibody 1A29

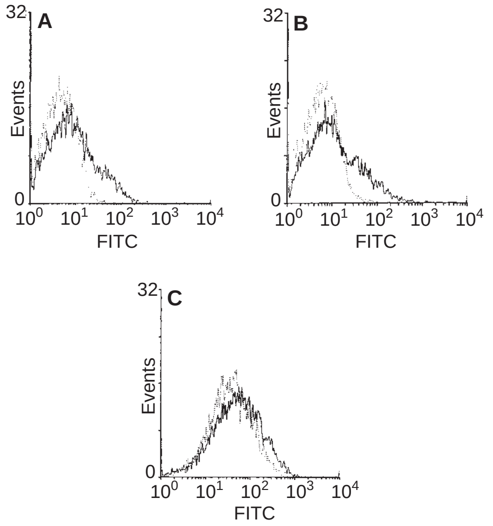

**Figure 4** Adhesion molecule expression of HUVEC as measured by flow cytometry after being exposed for 3 h to interactions with either resting PBMC (dotted line) or resting PBMC pre-incubated with BIS-1 F(ab′)$_2$ (solid line). For clarity, irrelevant antibody staining (MAb 1A29, MFI < 10), and expression of adhesion molecules by HUVEC that were not exposed to PBMC (identical to the expression by HUVEC exposed to PBMC) are not shown

### Involvement of soluble factors in BsMAb induced lymphocyte adhesion/tem

Tumour necrosis factor α (TNF-α) is a potent mediator of endothelial cell activation. We therefore investigated whether TNF release during the adhesion/tem of resting PBMC coated with BIS-1 F(ab′)$_2$ could be a cause of HUVEC activation. Supernatants of adhesion/tem assays were analysed in a bioassay using TNF-α-sensitive WEHI-164 cells (Morgan et al, 1991). Within the assay's concentration range of 1–32 pg ml$^{-1}$, no biologically active TNF-α could be detected (data not shown). We next performed adhesion/tem assays with BIS-1 F(ab′)$_2$-coated PBMC in the presence of 50 μg ml$^{-1}$ (0.3 μM) neutralizing anti-TNF-α antibody (MAb 61E71 (Leeuwenberg et al, 1988), kindly provided by Dr WA Buurman, Maastricht, The Netherlands). We hypothesized that by means of this, minute amounts of this cytokine that might have been produced locally at pharmacologically active concentrations enabling HUVEC activation, would be inactivated. The number of adherent/migrated cells did, however, not change in the presence of either anti-TNF-α or control antibody (data not shown). Also, no expression of transmembrane bound TNF-α, a possible mechanism by which the BIS-1 F(ab′)$_2$-coated lymphocytes could activate the endothelial cells during adhesion/tem without the release of soluble TNF-α (Lou et al, 1996), could be detected by FACS using PE-labelled anti-TNF-α (clone B-C7, kindly provided by Immuno Quality Products, Groningen, The Netherlands; data not shown).

Subsequently, we investigated whether any soluble factor released during the adhesion/tem assay could be the cause of the observed endothelial cell activation. Therefore, cell free medium harvested after the 3-h adhesion/tem assay was transferred to new, resting HUVEC monolayers. After 3 h incubation, HUVEC were phenotyped for the expression of E-selectin, VCAM-1 and ICAM-1. As can be seen from Figure 5, E-selectin did not become expressed when HUVEC were exposed to cell free medium of adhesion/tem assays of BIS-1 F(ab′)$_2$-coated PBMC. Similarly, no increase in VCAM-1 or ICAM-1 expression could be detected upon transferral and incubation of resting HUVEC with the cell free media (data not shown). Taken together, these data indicate that the observed endothelial cell activation was a result of direct contact between BIS-1 F(ab′)$_2$-coated PBMC and HUVEC.

### Effect of SHR-1 BsMAb on PBMC adhesion/tem

SHR-1 is a BsMAb developed for the treatment of CD19-positive tumours such as non-Hodgkin's lymphoma. SHR-1 consists of a CD3 recognizing fragment, like BIS-1 F(ab′)$_2$, and a CD19 recognizing fragment. After i.v. administration to patients with non-Hodgkin's lymphoma, SHR-1 also induced a rapid and transient lymphopenia (de Gast et al, 1995). To determine whether the effects observed with BIS-1 F(ab′)$_2$ were a BIS-1-specific or a more generalized phenomenon of CD3-directed BsMAbs, we studied the effects of SHR-1 binding to PBMC on PBMC–endothelial cell interactions. Under similar conditions as used with BIS-1 F(ab′)$_2$, SHR-1 binding to PBMC also resulted in an increased PBMC adhesion/tem (Figure 6). The effect of SHR-1 could be titrated down from a 6.7-fold increase in adhesion/tem after preincubation of resting PBMC at 1.0 μg ml$^{-1}$ SHR-1, to a 4.9-fold increase at 0.1 μg ml$^{-1}$, and a 2.3-fold increase at 0.01 μg ml$^{-1}$. Similarly, the effect of BIS-1 F(ab′)$_2$ could be titrated down to a 3.8-fold increase in adhesion/tem of resting PBMC incubated at 0.1 μg ml$^{-1}$ BIS-1 F(ab′)$_2$ and a 2.7-fold increase after

 © 2000 Cancer Research Campaign

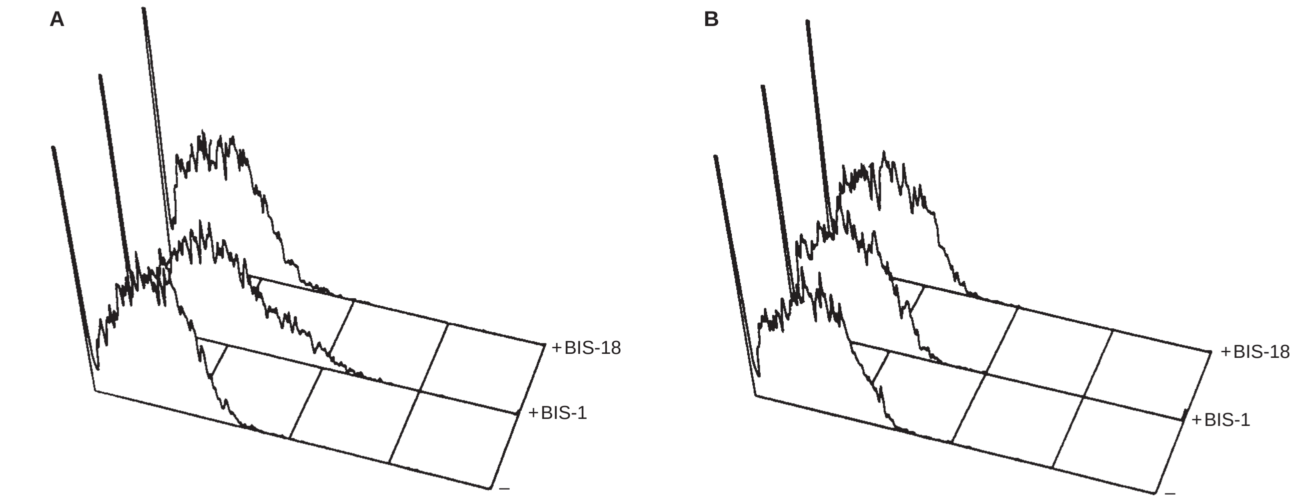

**Figure 5** (**A**) Flow cytometric analysis of E-selectin expression by HUVEC after facilitating adhesion/tem of resting PBMC or resting PBMC preincubated with either 1 μg $ml^{-1}$ BIS-1 F(ab′)$_2$ or BIS-18. (**B**) Flow cytometric analysis of E-selectin expression by HUVEC after 3 h exposure to cell free medium retrieved from adhesion/tem assays performed with resting PBMC or resting PBMC preincubated with either 1 μg $ml^{-1}$ BIS-1 F(ab′)$_2$ or BIS-18

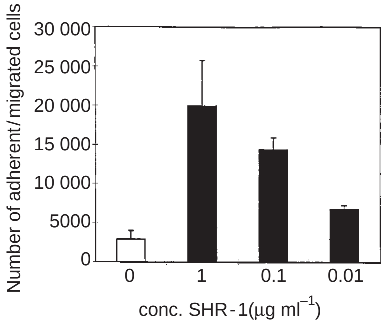

**Figure 6** Effect of SHR-1 binding to resting PBMC on adhesion/tem to/through resting HUVEC cultured on gelatin-coated wells as described in Materials and Methods. After pre-incubation of PBMC at RT for 45 min with 1, 0.1, 0.01 μg $ml^{-1}$ SHR-1, cells were subjected to the adhesion/tem assay as described in Materials and Methods incubation at 0.01 μg $ml^{-1}$. Phenotyping of HUVEC after facilitating SHR-1-coated PBMC adhesion/tem also revealed an increased expression of E-selectin, VCAM-1 and ICAM-1, and transferral of cell free assay medium, as described above, did not activate the HUVEC (data not shown). From these data, it is concluded that the effects of BIS-1 F(ab′)$_2$ binding to PBMC on PBMC–endothelial cell interactions are not restricted to the BsMAb BIS-1 F(ab′)$_2$ developed in our laboratory, but may well be a general characteristic of anti-CD3 fragment containing BsMAbs.

### DISCUSSION

The rapid and transient disappearance of lymphocytes from the peripheral blood of carcinoma patients upon i.v. BIS-1 F(ab′)$_2$ administration is at present not well understood. Possibly, the observed lymphopenia results from temporary lymphocyte margination. Eventually, increased margination may be followed by lymphocyte extravasation into organs and/or tumour tissue. Although CTLs need to leave the systemic circulation to become effective in solid tumour cell killing, for maximal anti-tumour effects an optimal redistribution of BIS-1 F(ab′)$_2$-coated CTLs to the tumour tissue is a prerequisite, and a generalized lymphocyte redistribution undesirable. For a lymphopenia to occur, the interaction between the lymphocytes and endothelial cells (of which the resting phenotype represents the majority of endothelial cells in the body) plays a central role. In this study, we investigated the effects of BIS-1 F(ab′)$_2$ binding to PBMC on generally accepted processes in leucocyte adhesion/tem, i.e. the expression of cell adhesion molecules and soluble factors leading to cell activation. We showed that BIS-1 F(ab′)$_2$ binding to resting PBMC resulted in increased PBMC–endothelial cell interactions. During the interaction between BIS-1 F(ab′)$_2$-coated PBMC and endothelial cells, the HUVEC became activated to express E-selectin and VCAM-1. This effect was most likely due to a direct interaction between the PBMC and the HUVEC, as soluble mediator(s) could not be demonstrated to be present in the assay medium. These effects were not BIS-1 F(ab′)$_2$-specific: the BsMAb SHR-1, developed for the treatment of CD19-positive tumours, exerted similar effects. Most likely, the increased PBMC–endothelial cell interactions were a CD3 binding related phenomenon, since the CD5-directed BsMAb BIS-18 did not affect PBMC adhesion/tem. Both antibody fragments present in BIS-1 F(ab′)$_2$ and SHR-1 recognize the epsilon chain of CD3, which is a signal transducing molecule. CD5, recognized by BIS-18, does however also lead to signal transduction in lymphocytes. Moreover, once the TcR/CD3 complex is engaged in signal transduction, the cytoplasmic domain of CD5 becomes rapidly phosphorylated, as are the TcR ζ chains (Gringhuis et al, 1998*a*, 1998*b*). The molecular basis for the observed differences of BIS-1 versus BIS-18 mediated effects on lymphocyte–endothelial cell interaction needs to be investigated further. In addition to the effects observed with BIS-1 F(ab′)$_2$, we found that binding of the parental CD3 recognizing MAb RIV9 induced PBMC adhesion/tem to a similar extent as BIS-1 F(ab′)$_2$ (unpublished data). Other CD3-directed MAbs also affected

PBMC–endothelial cell interactions, but experiments to relate MAb concentrations during PBMC preincubation and the extent of the effects on PBMC adhesion/tem need to be performed.

Recently, Buysmann et al showed that binding of the anti-CD3 MAb OKT3 to T lymphocytes induced a rapid adhesion of lymphocytes to resting endothelium (Buysmann et al, 1996). Comparison of both their and our study led us to conclude that different processes are most likely the basis for the observed effects. Whereas BIS-1 F(ab′)$_2$-coated PBMC only started to show an increased adhesion/tem after 15 min of incubation with HUVEC (unpublished data), OKT3 pre-incubation of T lymphocytes led to a maximum adhesion at 3 min of incubation followed by a rapid decline (Buysmann et al, 1996). Moreover, we were not able to block PBMC/BIS-1 F(ab′)$_2$ induced adhesion with TS1/18 antibody (anti-CD18; kindly provided by Dr N Oppenheimer-Marks, Dallas, TX, USA). In addition, neither VLA-4 or LFA-1/L15 neutralizing antibodies were able to specifically inhibit BIS-1 F(ab′)$_2$-induced PBMC adhesion/tem (data not shown). Our observation that preincubation of PBMC with either BIS-1 F(ab′)$_2$ or BIS-18 both induced VLA-4 and LFA-1 expression on PBMC, while only BIS-1 F(ab′)$_2$ binding led to an increase in PBMC–endothelial cell interaction strengthens the conclusion that the effects observed with BIS-1 F(ab′)$_2$ and OKT3 are based on a different mechanism(s). Possibly, L-selectin is involved in the effects observed, since L-selectin expression was lost upon BIS-1 F(ab′)$_2$ binding to PBMC, whereas no L-selectin modulation was seen upon OKT3 incubation (Buysmann et al, 1996).

The observation that adhesion/tem of BIS-1 F(ab′)$_2$- and SHR-1-coated PBMC leads to HUVEC activation is a novel finding. Recently, two other reports on cell–cell interaction-dependent endothelial cell activation were published. Sunderkotter et al found that trinitrochlorbenzene sensitized mouse T-cells were capable of inducing expression of E-selectin on microvascular endothelial cells after 4 h of co-culture. The mechanism by which such an activation occurred is unknown (Sunderkotter et al, 1996). Furthermore, Lou et al reported that stimulated T-cells were capable of activating endothelial cells upon direct cell contact. Membrane-associated TNF-α was partly responsible for this effect (Lou et al, 1996), but seems unlikely to be responsible for BsMAb/PBMC-induced HUVEC activation.

The data presented indicate the existence of lymphocyte–endothelial cell activation pathways of which the responsible molecular mechanism(s) remain(s) unclear. The fact is that BIS-1 F(ab′)$_2$ and SHR-1 binding to PBMC induces increased PBMC adhesion to resting endothelium which may (partly) explain the paradox of rapid lymphopenia in patients after start of BsMAb infusion without measurable TNF-α levels. Yet, extrapolation of these results to the clinical situation is difficult. In this study we have focused on resting PBMC, as the phenotype of PBMC of IL-2-treated patients at the start of BIS-1 F(ab′)$_2$ infusion is similar to that of the resting PBMC used with respect to the expression of L-selectin, VLA-4 and LFA-1 (G Molema, unpublished results). We also performed experiments with in vitro IL-2 and anti-CD3/IL-2-activated PBMC. The effects observed with BIS-1 F(ab′)$_2$-coated activated PBMC were similar to the effects seen with resting PBMC, although the extent of induction of adhesion/tem differed between PBMC populations (see Figure 2).

In a rat model of pulmonary metastases, we recently showed that treatment with a combination of rat IL-2 and rat CD3 × EGP-2-directed BsMAb induced a generalized leucocyte migration into kidney, liver and lung parenchyma. This effect coincided with an increased endothelial VCAM-1 expression in these organs. As a selective antibody for rat E-selectin is not available, E-selectin expression could not be immunohistochemically analysed yet. Furthermore, PBMC isolated from IL-2-treated cancer patients exerted an increased adhesion/tem in vitro when pre-incubated with BIS-1 F(ab′)$_2$ (G Molema, unpublished data). These data indicate that in vivo activated leucocytes do respond to CD3-directed BsMAb in a similar way as described in this study.

Our observations warrant investigations into the occurrence of effects described in this study in patients receiving i.v. BIS-1 F(ab′)$_2$, as it may explain the lack of redistribution of effective amounts of CTL to the solid tumour tissue. Furthermore, comparison of the effects of other CD3-directed MAbs or BsMAbs on PBMC adhesion/tem may provide a tool to differentiate between antibodies with respect to their desired therapeutic application. BsMAbs and newly developed diabodies (Helfrich et al, 1998), are developed for redirecting cytolytic activity towards tumour cells. Redistribution of lymphocytes to other sites than the tumour is therefore undesirable.

The observation that the CD5-directed BsMAb BIS-18 was devoid of an effect on PBMC adhesion/tem may be exploited for the development of more selective targeting entities to, e.g. tumour vasculature-specific antigens (Molema et al, 1997, 1998*b*). After distribution to the tumour vascular endothelium, lymphocyte extravasation towards the tumour cells may be accomplished by subsequent activation. Furthermore, to redirect the cytolytic activity against the tumour vascular endothelial cells, selective redistribution of CTLs by means of a CD5 × tumour endothelial antigen-directed BsMAb may be envisioned without the occurrence of a generalized loss of the immunocompetent cells to other sites in the body.

### ACKNOWLEDGEMENTS

Roelke Emmens and Henk Moorlag are acknowledged for performing phenotyping of the cells and HUVEC isolation and culture, and Geert Mesander for assistance during flow cytometric analysis. Annie Bakker is acknowledged for the preparation of the BIS-18 bispecific antibody. Diane Bouis is thanked for performing adhesion/tem assays. This research was financially supported by the Royal Netherlands Academy of Arts and Sciences (KNAW; GM and JWCT) and the Dutch Cancer Foundation (BJK).

### REFERENCES

Buysmann S, Bemelman FJ, Schellekens PT, Van Kooyk Y, Figdor CG and ten Berge IJ (1996) Activation and increased expression of adhesion molecules on peripheral blood lymphocytes is a mechanism for the immediate lymphocytopenia after administration of OKT3. *Blood* **87**: 404–411

Clark M, Bindon C, Dyer M, Friend P, Hale G, Cobbold S, Calne R and Waldmann H (1989) The improved lytic function and in vivo efficacy of monovalent monoclonal CD3 antibodies. *Eur J Immunol* **19**: 381–388

de Gast GC, Haagen IA, van Houten AA, Klein SC, Duits AJ, de Weger RA, Vroom TM, Clark MR, Phillips J, van Dijk AJ, et al (1995) CD8 T cell activation after intravenous administration of CD3 × CD19 bispecific antibody in patients with non-Hodgkin lymphoma. *Cancer Immunol Immunother* **40**: 390–396

de Leij L, Helfrich W, Stein R and Mattes MJ (1994) SCLC-cluster-2 antibodies detect the pancarcinoma/epithelial glycoprotein EGP-2. *Int J Cancer* **8**: 60–63

Demanet C, Brissinck J, De Jonge J and Thielemans K (1996) Bispecific antibody-mediated immunotherapy of the BCL1 lymphoma: increased efficacy with multiple injections and CD28-induced costimulation. *Blood* **87**: 4390–4398

 © 2000 Cancer Research Campaign

Gringhuis SI, de Leij L, Wayman GA, Tokumitsu H and Vellenga E (1998*a*) The $Ca^{2+}$/calmodulin-dependent kinase type IV is involved in the CD5-mediated signalling pathway in human T lymphocytes. *J Biol Chem* **272**: 31809–31820
Gringhuis SI, de Leij LFMH, Coffer PJ and Vellenga E (1998*b*) Signalling through CD5 activates a pathway involving phosphatidylinositol 3-kinase, VaV, and RacI in human mature T lymphocytes. *Mol Cell Biol* **18**: 1725–1735
Helfrich W, Kroesen BJ, Roovers RC, Westers L, Molema G, Hoogenboom HR and de Leij L (1998) Construction and characterization of a bispecific diabody for retargeting T-cells to human carcinomas. *Int J Cancer* **76**: 232–239
Janssen RAJ, Kroesen BJ, Buter J, Mesander G, Sleijfer DT, The TH, Mulder NH and de Leij L (1995) Immunomodulatory effects of intravenous BIS-1 F(ab′)2 administration in renal cancer patients. *Br J Cancer* **72**: 795–799
Kroesen BJ, ter Haar A, Spakman H, Willemse P, Sleijfer DT, de Vries EGE, Mulder NH, Berendsen HH, Limburg PC, The TH and de Leij L (1993) Local antitumour treatment in carcinoma patients with bispecific-monoclonal-antibody-redirected T cells. *Cancer Immunol Immunother* **37**: 400–407
Kroesen BJ, Buter J, Sleijfer DT, Janssen RAJ, van der Graaf WTA, The TH, de Leij L and Mulder NH (1994) Phase I study of intravenously applied bispecific antibody in renal cell cancer patients receiving subcutaneous interleukin 2. *Br J Cancer* **70**: 652–661
Kroesen BJ, Bakker A, Van Lier RAW, The HT and de Leij L (1995) Bispecific antibody-mediated target cell-specific costimulation of resting T cells via CD5 and CD28. *Cancer Res* **55**: 4409–4415
Leeuwenberg JF, Van Damme J, Meager T, Jeunhomme TM and Buurman WA (1988) Effects of tumor necrosis factor on the interferon-gamma-induced major histocompatibility complex class II antigen expression by human endothelial cells. *Eur J Immunol* **18**: 1469–1472
Lou J, Dayer J, Grau G and Burger D (1996) Direct cell/cell contact with stimulated T lymphocytes induces the expression of cell adhesion molecules and cytokines by human brain microvascular endothelial cells. *Eur J Immunol* **26:** 3107–3113
Masinovsky B, Urdal D and Gallatin WM (1990) IL-4 acts synregistically with IL-1beta to promote lymphocyte adhesion to microvascular endothelium by induction of vascular cell adhesion molecule-1. *J Immunol* **145**: 2886–2895.
Molema G, de Leij LFMH and Meijer DKF (1997) Tumor vascular endothelium: barrier or target in tumor directed drug delivery and immunotherapy. *Pharm Res* **14**: 2–10.
Molema G, Mesander G, Kroesen BJ, Helfrich W, Meijer DKF and de Leij LFMH (1998*a*) Analysis of in vitro lymphocyte adhesion and transendothelial migration by fluorescent beads based flow cytometric cell counting. *Cytometry* **32**: 37–43.
Molema G, Meijer DKF and de Leij LFMH (1998*b*) Tumor vasculature targeted therapies: getting the players organized. *Biochem Pharmacol* **55**: 1939–1945.
Morgan CD, Mills KC, Lefkowitz DL and Lefkowitz SS (1991) An improved colorimetric assay for tumor necrosis factor using WEHI 164 cells cultured on novel microtiter plates. *J Immunol Methods* **145**: 259–262.
Mulder AB, Hegge Paping KS, Magielse CP, Blom NR, Smit JW, van der Meer J, Hallie MR and Bom VJ (1994) Tumor necrosis factor alpha-induced endothelial tissue factor is located on the cell surface rather than in the subendothelial matrix. *Blood* **84**: 1559–1566.
Oppenheimer-Marks N, Davis LS, Tompkins Bogue D, Ramberg J and Lipsky PE (1991) Differential utilization of ICAM-1 and VCAM-1 during the adhesion and transendothelial migration of human T lymphocytes. *J Immunol* **147**: 2913–2921.
Renner C, Bauer S, Sahin U, Jung W, van Lier R, Jacobs G, Held G and Pfreundschuh M (1996) Cure of disseminated xenografted human Hodgkin's tumors by bispecific monoclonal antibodies and human T cells: the role of human T-cell subsets in a preclinical model. *Blood* **87**: 2930–2937.
Springer TA (1994) Traffic signals for lymphocyte recirculation and leukocyte emigration: the multistep paradigm. *Cell* **76**: 301–314
Sunderkotter C, Steinbrink K, Henseleit U, Bosse R, Schwarz A, Vestweber D and Sorg C (1996) Activated T cells induce expression of E-selectin in vitro and in an antigen-dependent manner in vivo. *Eur J Immunol* **26**: 1571–1579
Tax WJ, Willems HW, Reekers PP, Capel PJ and Koene RA (1983) Polymorphism in mitogenic effect of IgG1 monoclonal antibodies against T3 antigen on human T cells. *Nature* **304**: 445–447
Thornhill MH, Kyan-Aung U and Haskard DO (1990) IL-4 increases human endothelial cell adhesiveness for T cells but nor for neutrophils. *J Immunol* **144**: 3060–3065
Van Kooyk Y, Weder P, Hogervorst F, Verhoeven AJ, van Seventer G, te Velde AA, Borst J, Keizer GD and Figdor CG (1991) Activation of LFA-1 through a $Ca^{2(+)}$-dependent epitope stimulates lymphocyte adhesion. *J Cell Biol* **112:** 345–354

© 2000 Cancer Research Campaign 

## References

[PDF_00741] Buysmann S., Bemelman F. J., Schellekens P. T., van Kooyk Y., Figdor C. G., ten Berge I. J. (1996). Activation and increased expression of adhesion molecules on peripheral blood lymphocytes is a mechanism for the immediate lymphocytopenia after administration of OKT3.. Blood.

[PDF_00744] Clark M., Bindon C., Dyer M., Friend P., Hale G., Cobbold S., Calne R., Waldmann H. (1989). The improved lytic function and in vivo efficacy of monovalent monoclonal CD3 antibodies.. Eur J Immunol.

[PDF_00751] De Leij L., Helrich W., Stein R., Mattes M. J. (1994). SCLC-cluster-2 antibodies detect the pancarcinoma/epithelial glycoprotein EGP-2.. Int J Cancer Suppl.

[PDF_00753] Demanet C., Brissinck J., De Jonge J., Thielemans K. (1996). Bispecific antibody-mediated immunotherapy of the BCL1 lymphoma: increased efficacy with multiple injections and CD28-induced costimulation.. Blood.

[PDF_00763] Gringhuis S. I., de Leij L. F., Coffer P. J., Vellenga E. (1998). Signaling through CD5 activates a pathway involving phosphatidylinositol 3-kinase, Vav, and Rac1 in human mature T lymphocytes.. Mol Cell Biol.

[PDF_00760] Gringhuis S. I., de Leij L. F., Wayman G. A., Tokumitsu H., Vellenga E. (1997). The Ca2+/calmodulin-dependent kinase type IV is involved in the CD5-mediated signaling pathway in human T lymphocytes.. J Biol Chem.

[PDF_00766] Helfrich W., Kroesen B. J., Roovers R. C., Westers L., Molema G., Hoogenboom H. R., de Leij L. (1998). Construction and characterization of a bispecific diabody for retargeting T cells to human carcinomas.. Int J Cancer.

[PDF_00769] Janssen R. A., Kroesen B. J., Buter J., Mesander G., Sleijfer D. T., The T. H., Mulder N. H., de Leij L. (1995). Immunomodulatory effects of intravenous BIS-1 F(ab')2 administration in renal cell cancer patients.. Br J Cancer.

[PDF_00780] Kroesen B. J., Bakker A., van Lier R. A., The H. T., de Leij L. (1995). Bispecific antibody-mediated target cell-specific costimulation of resting T cells via CD5 and CD28.. Cancer Res.

[PDF_00776] Kroesen B. J., Buter J., Sleijfer D. T., Janssen R. A., van der Graaf W. T., The T. H., de Leij L., Mulder N. H. (1994). Phase I study of intravenously applied bispecific antibody in renal cell cancer patients receiving subcutaneous interleukin 2.. Br J Cancer.

[PDF_00772] Kroesen B. J., ter Haar A., Spakman H., Willemse P., Sleijfer D. T., de Vries E. G., Mulder N. H., Berendsen H. H., Limburg P. C., The T. H. (1993). Local antitumour treatment in carcinoma patients with bispecific-monoclonal-antibody-redirected T cells.. Cancer Immunol Immunother.

[PDF_00783] Leeuwenberg J. F., Van Damme J., Meager T., Jeunhomme T. M., Buurman W. A. (1988). Effects of tumor necrosis factor on the interferon-gamma-induced major histocompatibility complex class II antigen expression by human endothelial cells.. Eur J Immunol.

[PDF_00787] Lou J., Dayer J. M., Grau G. E., Burger D. (1996). Direct cell/cell contact with stimulated T lymphocytes induces the expression of cell adhesion molecules and cytokines by human brain microvascular endothelial cells.. Eur J Immunol.

[PDF_00790] Masinovsky B., Urdal D., Gallatin W. M. (1990). IL-4 acts synergistically with IL-1 beta to promote lymphocyte adhesion to microvascular endothelium by induction of vascular cell adhesion molecule-1.. J Immunol.

[PDF_00800] Molema G., Meijer D. K., de Leij L. F. (1998). Tumor vasculature targeted therapies: getting the players organized.. Biochem Pharmacol.

[PDF_00796] Molema G., Mesander G., Kroesen B. J., Helfrich W., Meijer D. K., de Leij L. F. (1998). Analysis of in vitro lymphocyte adhesion and transendothelial migration by fluorescent-beads-based flow cytometric cell counting.. Cytometry.

[PDF_00793] Molema G., de Leij L. F., Meijer D. K. (1997). Tumor vascular endothelium: barrier or target in tumor directed drug delivery and immunotherapy.. Pharm Res.

[PDF_00802] Morgan C. D., Mills K. C., Lefkowitz D. L., Lefkowitz S. S. (1991). An improved colorimetric assay for tumor necrosis factor using WEHI 164 cells cultured on novel microtiter plates.. J Immunol Methods.

[PDF_00805] Mulder A. B., Hegge-Paping K. S., Magielse C. P., Blom N. R., Smit J. W., van der Meer J., Hallie M. R., Bom V. J. (1994). Tumor necrosis factor alpha-induced endothelial tissue factor is located on the cell surface rather than in the subendothelial matrix.. Blood.

[PDF_00809] Oppenheimer-Marks N., Davis L. S., Bogue D. T., Ramberg J., Lipsky P. E. (1991). Differential utilization of ICAM-1 and VCAM-1 during the adhesion and transendothelial migration of human T lymphocytes.. J Immunol.

[PDF_00813] Renner C., Bauer S., Sahin U., Jung W., van Lier R., Jacobs G., Held G., Pfreundschuh M. (1996). Cure of disseminated xenografted human Hodgkin's tumors by bispecific monoclonal antibodies and human T cells: the role of human T-cell subsets in a preclinical model.. Blood.

[PDF_00817] Springer T. A. (1994). Traffic signals for lymphocyte recirculation and leukocyte emigration: the multistep paradigm.. Cell.

[PDF_00819] Sunderkötter C., Steinbrink K., Henseleit U., Bosse R., Schwarz A., Vestweber D., Sorg C. (1996). Activated T cells induce expression of E-selectin in vitro and in an antigen-dependent manner in vivo.. Eur J Immunol.

[PDF_00822] Tax W. J., Willems H. W., Reekers P. P., Capel P. J., Koene R. A. (1983). Polymorphism in mitogenic effect of IgG1 monoclonal antibodies against T3 antigen on human T cells.. Nature.

[PDF_00825] Thornhill M. H., Kyan-Aung U., Haskard D. O. (1990). IL-4 increases human endothelial cell adhesiveness for T cells but not for neutrophils.. J Immunol.

[PDF_00747] de Gast G. C., Haagen I. A., van Houten A. A., Klein S. C., Duits A. J., de Weger R. A., Vroom T. M., Clark M. R., Phillips J., van Dijk A. J. (1995). CD8 T cell activation after intravenous administration of CD3 x CD19 bispecific antibody in patients with non-Hodgkin lymphoma.. Cancer Immunol Immunother.

[PDF_00829] van Kooyk Y., Weder P., Hogervorst F., Verhoeven A. J., van Seventer G., te Velde A. A., Borst J., Keizer G. D., Figdor C. G. (1991). Activation of LFA-1 through a Ca2(+)-dependent epitope stimulates lymphocyte adhesion.. J Cell Biol.

